# Mitochondrial DNA analysis of field populations of *Helicoverpa armigera *(Lepidoptera: Noctuidae) and of its relationship to *H. zea*

**DOI:** 10.1186/1471-2148-7-117

**Published:** 2007-07-14

**Authors:** Gajanan T Behere, Wee Tek Tay, Derek A Russell, David G Heckel, Belinda R Appleton, Keshav R Kranthi, Philip Batterham

**Affiliations:** 1 Centre for Environmental Stress and Adaptation Research, Department of Genetics, Bio21 Institute of Molecular Science and Biotechnology, The University of Melbourne, Parkville 3010, Australia; 2 Natural Resources Institute, University of Greenwich, Chatham Maritime, Kent, Me4 4TB, UK; 3 Department of Entomology, Max-Planck Institute for Chemical Ecology, Beutenberg Campus, Hans-Knöll-Straße 8, Jena D-07745, Germany; 4 Department of Genetics, The University of Melbourne, Parkville 3010, Australia; 5 Central Institute for Cotton Research, Post Bag No 2, Shankar Nagar P.O., Nagpur 440010, India

## Abstract

**Background:**

*Helicoverpa armigera *and *H. zea *are amongst the most significant polyphagous pest lepidopteran species in the Old and New Worlds respectively. Separation of *H. armigera *and *H. zea *is difficult and is usually only achieved through morphological differences in the genitalia. They are capable of interbreeding to produce fertile offspring. The single species status of *H. armigera *has been doubted, due to its wide distribution and plant host range across the Old World. This study explores the global genetic diversity of *H. armigera *and its evolutionary relationship to *H zea*.

**Results:**

We obtained partial (511 bp) mitochondrial DNA (mtDNA) Cytochrome Oxidase-I (COI) sequences for 249 individuals of *H. armigera *sampled from Australia, Burkina Faso, Uganda, China, India and Pakistan which were associated with various host plants. Single nucleotide polymorphisms (SNPs) within the partial COI gene differentiated *H. armigera *populations into 33 mtDNA haplotypes. Shared haplotypes between continents, low *F*-statistic values and low nucleotide diversity between countries (0.0017 – 0.0038) suggests high mobility in this pest. Phylogenetic analysis of four major *Helicoverpa *pest species indicates that *H*. *punctigera *is basal to *H*. *assulta*, which is in turn basal to *H*. *armigera *and *H*. *zea*. Samples from North and South America suggest that *H. zea *is also a single species across its distribution. Our data reveal short genetic distances between *H. armigera *and *H. zea *which seem to have been established via a founder event from *H. armigera *stock at around 1.5 million years ago.

**Conclusion:**

Our mitochondrial DNA sequence data supports the single species status of *H. armigera *across Africa, Asia and Australia. The evidence for inter-continental gene flow observed in this study is consistent with published evidence of the capacity of this species to migrate over long distances. The finding of high genetic similarity between Old World *H. armigera *and New World *H. zea *emphasises the need to consider work on both pests when building pest management strategies for either.

## Background

The genus *Helicoverpa *(Hardwick 1965) is a group of 18 species [1] which includes some of the most devastating agricultural lepidopteran pest species, with *H. armigera *(Hübner) and *H. zea *(Boddie) being the dominant pest species in the Old World and New World respectively. A few other species in the genus *Helicoverpa *are pests of a range of crops but they are either limited in host plant range or are geographically restricted [1]. This includes *H. assulta *(Guenée) which feeds only on Solanaceae, and is endemic to Asia, Africa and Australia [2], and *H. punctigera *(Wallengren) which is polyphagous and endemic to Australia [3]. The majority of *Helicoverpa *species are oligophagous and are not considered as major agricultural pests.

Until the work of Hardwick [4]*H. zea *and *H. armigera *were considered conspecific [5] within the genus *Heliothis *Oschenheimer [6] as *Heliothis armigera*. Hardwick [4] incorporated results from extensive morphological, rearing and hybridization studies, arranged these moths into a postulated evolutionary sequence of species groups, and at the same time resolved the taxonomic position of *H. armigera *and *H. zea *as separate species within the new *Helicoverpa *genus, Matthews [2] confirmed the criteria used by Hardwick [4] for the *Helicoverpa *genus, developing methods to inflate the helical bladder-like appendages in this genus. Morphological characters and allozyme-based phylogeny suggested that *H. punctigera *is basal to *H. assulta *[1]. The evolutionary relationship between *H. armigera *and *H. zea *was uncertain and they were considered monophyletic, sharing a common ancestor with *H. assulta *[1]. Allozyme studies revealed similarly large heterozygosities in *H. armigera *and *H. punctigera *but with *H. zea *displaying 61% less mean heterozygosity, and Mallet et al. [7] suggested that *H. zea *evolved from a small founding population of *H. armigera *(or of their joint common ancestor). Accurate demarcation of species boundaries and their distributions is essential for understanding pest demography. Apart from the work of Matthews [2] which was confined to Australia, there is a lack of firm systematic foundation in this genus and specifically that of important *Helicoverpa *pest species such as *H. armigera *and *H. zea*. This represents a significant handicap for basic and applied research into these two important pest species.

Larvae of *H. armigera *and *H. zea *are highly polyphagous. They possess the ability to enter diapause as pupae and are known to develop high levels of insecticide resistance [8-11]. Adult moths also demonstrate high mobility (capable of travelling over 1,000 km) and fecundity (individual females are capable of laying up to 3,000 eggs) [3]. Population genetic studies of *H. armigera *have been conducted in different regions of the world using different genetic marker systems [12-18]. Varying results from these studies reflect interactions between different agricultural practices and the life history of the pest species, and the nature of the different genetic marker systems applied. In Australia, studies based on isozymes [12], mitochondrial DNA (mtDNA) control region [13] and the sodium channel gene [14] suggest large effective population size in *H. armigera*. However, microsatellite studies of Australian *H. armigera *populations suggested monthly genetic shifts, highly variable gene flow between populations between years, and limited moth movements which varied between and within seasons [15,16]. Results from these microsatellite DNA studies contrast with studies carried out in eastern Mediterranean populations using random amplified polymorphic DNA [17], and in African and European samples using isozymes [18]. These studies found little genetic variation between widely separated populations, supporting the idea that extensive long distance migration occurred in *H. armigera*. Low genetic differentiation in the related long-range migratory pest *Heliothis virescens *across the cotton belt within the United States of America has also been reported [19].

Despite intense agricultural interest in *H. armigera *as a polyphagous pest, very little systematic research has been conducted to resolve the question of the existence of sub-species at the local or global level. Based on taxonomic data, Paterson [20] raised doubts as to whether *H*. *armigera *constituted a single genetic species or formed a complex of cryptic species over its geographic range. Although the existence of cryptic *H. armigera *species has not been disproved, mating experiments that involved *H. armigera *from different regions of the Old World strongly supported its single species status [21]. In India, various reports suggested that *H. armigera *could be categorized into races, based on their host-feeding preferences, and that these races do not interbreed freely [22,23]. Kranthi et al. [24] reported variable metabolic mechanisms mediating pyrethroid resistance, with the shift from MFO-mediated pyrethroid resistance to an esterase-mediated mechanism during mid October in central India, which might have been related to different *H. armigera *populations being sampled at these times from different crops. Differential responses of *H. armigera *populations to pheromone [25] and parasitoids [26] were also reported in India. It has been suggested that independent evolution of lineages may be demarcated by food plant differentiation, facilitating the development of host races or host differentiated species [27,28]. "Cotton" and "non cotton" field races of *H. armigera *have also been generated through laboratory selection of field-collected insects on cotton fruit buds (squares). These races were characterized by differing (though overlapping) cornutal spine number on the male aedeagus [29].

To date, no molecular studies have assessed phylogeographic patterns among worldwide populations of *H. armigera*. Analysis of mtDNA has provided valuable insights into understanding natural genetic diversity and population structures in other organisms [30]. It is clear that both demographic and bio-geographic forces shape the depth and distribution of lineages in a phylogenetic tree, while selecting the correct loci in constructing a phylogeny is of equal importance due to differing evolutionary rates among different DNA regions, and within and between different loci (eg, nuclear versus mtDNA loci). For example, interpreting mtDNA phylogeny should proceed with caution because this locus is highly prone to selective sweeps due to its single locus nature and the general lack of recombination. Furthermore, inherited symbionts such as Wolbachia and other bacteria may also cause maternal cytotype sweeps [31]. Nevertheless, the mtDNA genes, especially that of the Cytochrome Oxidase sub-unit I (COI) gene have been used extensively in phylogenetic studies due to the ease of primer design and its range of phylogenetic signal. The rate of evolution in this gene is also sufficiently rapid to allow the discrimination at the species level and the identification of cryptic species [32], and has been used in establishing host plant associated genetic differentiation [33]. This study therefore aims to provide the first broad scale screening of mtDNA variation in *H. armigera *globally. The genetic information collected is used to address the questions of single species status and of host races, and to infer phylogenetic relationships amongst the four pest *Helicoverpa *species.

## Results

### PCR amplification and sequence analysis

**Figure 1:**
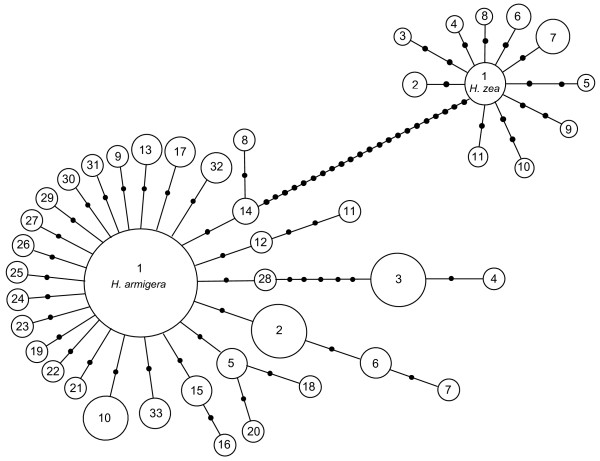
Haplotype network based on partial mtDNA COI (511 bp) of *H. armigera*, sampled from Australia, Burkina Faso, Uganda, China, India and Pakistan. Each haplotype is represented by a circle, and is identified by a number 1–33. Haplotype 1 included 156 individuals; haplotypes 2, 3, 5 and 10 have 17, 15, 5 and 10 individuals respectively. Haplotypes 6, 32 and 33 each have 4 individuals. Haplotypes 13 and 17 each has 3 individuals, and Haplotype 4, 11, 14, 15 and 19 each have 2 individuals. All remaining haplotypes have 1 individual each. Each base change involved in the differentiation between haplotypes is represented by a solid circle.

Preliminary population sequence survey of 809 bp mtDNA region (see Methods) indicated the partial COI gene at nucleotide positions 1 to 511 as being the most informative region for our *H. armigera *and *H. zea *population study. The 62 randomly sampled *H. armigera *and 11 *H. zea *haplotype representative samples all possessed identical tRNA-Leu gene sequence to the deposited *H. armigera *tRNA-Leu sequence (DQ059302). In the 62 *H. armigera *samples, two transitional mutations (G/A and C/T) were detected. The G/A mutation involved one individual each from Burkina Faso and Pakistan that belonged to the same mtDNA haplotype (Harm1). The C/T base change involved an Australian (previously Harm2) and a Pakistan (previously Harm3) sample. There were four C/T transitional mutations from nucleotide positions 512 to 809 between the 11 *H. zea *and the 62 *H. armigera *samples. These four mutations increased the number of steps by one between haplotype Hzea-1 and Hzea-7, and the number of steps by three in the haplotype network between *H. armigera *and *H. zea *(Fig 1). All transitional mutations between nucleotide 512 to 809 were parsimoniously uninformative at the intra- and inter-species levels in *H. armigera *and *H. zea*. The mtDNA region from nucleotide position 512 to 809 was therefore excluded in analyses of all remaining samples.

PCR amplification of the informative partial mtDNA COI fragments for all individuals of *H. armigera*, *H. zea, H. punctigera, H. assulta *and *Heliothis virescens *gave PCR products of the expected size. A final 511 base pair (bp) sequence (nucleotide position 1 to 511) was analysed, following trimming of invalid end sequences and sequence alignment. The COI sequences showed no ambiguity and no premature stop codons. A total of 32 SNPs were identified from the 511 bp partial COI region in 249 *H. armigera *samples resulting in 33 mtDNA haplotypes. A total of 29 synonymous and 3 non-synonymous substitutions were detected in these partial COI sequences. Multiple amino acid sequence alignments revealed that the three non-synonymous changes are in haplotype Harm-11 (Glycine to Serine); Harm-13 (Isoleucine to Phenylalaine) and Harm-26 (Tyrosine to Cysteine). Amino acid substitutions in Harm-13 and Harm-26 resulted in the replacement of one hydrophobic amino acid with another, while in Harm-11 the amino acid substitution occurred between hydrophobic and polar groups. Of the 32 variable sites identified, 15 sites were parsimony informative and 17 were non-informative.

### Haplotypes

The most prevalent haplotype found in all countries was designated Harm-1. Harm-4, found in Uganda and Australia, was the most diverged haplotype with seven mutation steps from the major haplotype Harm-1 (Fig. 1). Haplotypes Harm-1, 2, 3 and 10 formed the major haplotypes commonly found in all countries except Uganda (Harm-10) and Pakistan (Harm-2, 3 and 10) (Table 1). The frequencies of unique haplotypes were comparatively low within individual countries (Table 2). The haplotype network revealed no major groupings of *H. armigera *haplotypes according to either host plants or geographical clade (Fig. 1 and Table 1). A total of 11 haplotypes were identified from 64 *H. zea *individuals sampled from Northern (North Carolina and New York) and South America (Brazil). Haplotypes Hzea-1, 2, 6 and 7 were found in both North and South American continents, while the remaining haplotypes were unique to either the North or South American populations. The *H. zea *haplotype network is most parsimoniously linked to the *H. armigera *clade through Harm-14 found in Burkina Faso (Fig. 1). All mtDNA COI haplotypes identified in this study have been deposited in GenBank (EF116226-EF116274).

**Table 1:** Sample list, countries of origin, host plants, number of individuals and collection dates of *Helicoverpa *species used in current study.

**Countries**	**Locations**	**Source**	**Collection dates**	**Haplotypes**
***Helicoverpa armigera***				
India	Mansa (5)	Chickpea	Jan.2005	1 & 24
	Bhatinda (5)	Chickpea	Jan.2005	1,17,19 & 23
	Abohar (5)	Chickpea	Jan.2005	1 & 10
	Yavatmal (10)	Egg Plant	Jul.2005	1,3,15 & 17
	Hingoli (5)	Cotton	Nov.2004	1 & 10
	Nagpur (11)	Pheromone(P1)	Jan.2005	1,3 & 27
	Prakasam (6)	Cotton	Dec.2004	1
	Coimbatore (22)	Pigeonpea	Jan.2005	1,3,6,9 & 10
	Karimnagar (10)	Cotton	Oct.2005	1,2 & 32
	Warangal (11)	Cotton	Oct.2005	1,32 & 33
Burkina Faso	Kenedougou (35)	Tomato	Mar.2003	1,2,3,10,14,16 & 17
Uganda	Kampala (24)	Cotton	Nov.2005	1,2,3,4,11,12,19,25 & 26
Australia	Orbost (24)	Corn	Apr.2005	1,2,3,5,13,29 & 31
	Dalmore (22)	Corn	Apr.2005	1,2,4,5,8 & 13
	Werribee (10)	Pheromone (P2)	Jan.2001	1,2,3,6,10,18,20,28 & 30
China	Shandong (34)	Cotton	Feb.2005	1,2,3,6,10,15,21 & 22
Pakistan	Multan (10)	Cotton	Nov.2004	1,6 & 7
***Helicoverpa zea***				
USA	North Carolina (14)	Cotton	2002	Hzea-1 & 2
	New York (20)	Pheromone	Dec.2005	Hzea-1,2,3,4,5,6 & 7
Brazil	Primavera Do Leste (30)	Corn	Apr.2006	Hzea-1,2,6,7,8,9,10 & 11
***Helicoverpa assulta***				
India	Nagpur (5)	Dhatura	Jul.2005	
***Helicoverpa. Punctigera***				
Australia	Werribee (5)	Pheromone	Mar.2005	

P1 and P2 represent moths collected by pheromone when surrounding crops were cotton and corn respectively. Numbers in parenthesis indicates number of individuals sequenced. Northern Indian samples are: Mansa, Bhatinda, Abohar; Central Indian samples are: Yavatmal, Hingoli, Nagpur; and Southern Indian samples are Prakasam, Coimbatore, Karimnagar and Warangal.

**Table 2:** Number of unique and shared haplotypes identified in different countries. Numbers in parenthesis indicates the frequencies of haplotypes within countries.

Countries	Number of haplotypes	Number of unique haplotypes	Number of shared haplotypes
Australia	14	9 (0.27)	5 (0.73)
China	8	2 (0.06)	6 (0.94)
India	14	6 (0.13)	8 (0.87)
Pakistan	3	1 (0.10)	2 (0.90)
Burkina Faso	7	2 (0.09)	5 (0.91)
Uganda	9	4 (0.21)	5 (0.79)
**World**	**33**	**24 (0.15)**	**9 (0.85)**

### F-statistics (*F*_st_) and Analysis of Molecular Variance (AMOVA)

Because of the generally small sample sizes in the individual collections from the Indian sub-continent, populations were grouped as Central (Nagpur, Yavatmal and Hingoli), North (Abohar, Bhatinda, Mansa and Pakistan) and South (Prakasam, Karimnagar, Warangal and Coimbatore) in *F*-statistics analysis which showed significant gene flow between these three regions (*F*_st _= 0.07). Analysis of Molecular Variance detected no genetic structure at various hierarchical levels (Table 3), with 96.88% of variation accounted for at the within population (ie, within country) level, with only 2.15% variation observed among groups (ie, Asian, African and Australian continents). Pairwise *F*_st _values (when considering individual countries as separate populations) were low in the study samples, and ranged from approximately 0 to 0.06. The observed nucleotide diversity between countries was also very low and ranged from 0.0017 – 0.0039 (Table 4).

**Table 3:** Hierarchical analysis and associated probabilities of among populations and groups.

Hierarchical levels	Variance	Percentage of variation	Fixation indices	P value
Among groups	0.00649	2.15	0.02146 F_CT_	NS
Among populations within groups	0.00292	0.97	0.00987 F_SC_	NS
Within populations	0.2929	96.88	0.03112 F_ST_	<0.01

Fixation indices for: among groups (F_CT_), taken as the three continents of Asia (India, Pakistan and China), Africa (Burkina Faso and Uganda) and Australia (Orbost, Dalmore and Werribee); among populations within groups (F_SC_), and within populations (F_ST_). Non significant P value (NS). A total of 10 populations from India were pooled as no significant differences were found between populations.

**Table 4:** Comparison of *H. armigera *mtDNA partial COI nucleotide diversity (π) and haplotype diversity (*h*) between different countries.

Countries	Nucleotide diversity	Haplotype diversity
Australia	0.0029 ± 0.0020	0.7338 ± 0.0546
China	0.0022 ± 0.0016	0.4617 ± 0.1054
India	0.0032 ± 0.0021	0.5865 ± 0.0594
Pakistan	0.0017 ± 0.0015	0.3778 ± 0.1813
Burkina Faso	0.0017 ± 0.0013	0.4874 ± 0.1010
Uganda	0.0038 ± 0.0025	0.6630 ± 0.1075

### Phylogenetic inference and genetic distance

**Figure 2:**
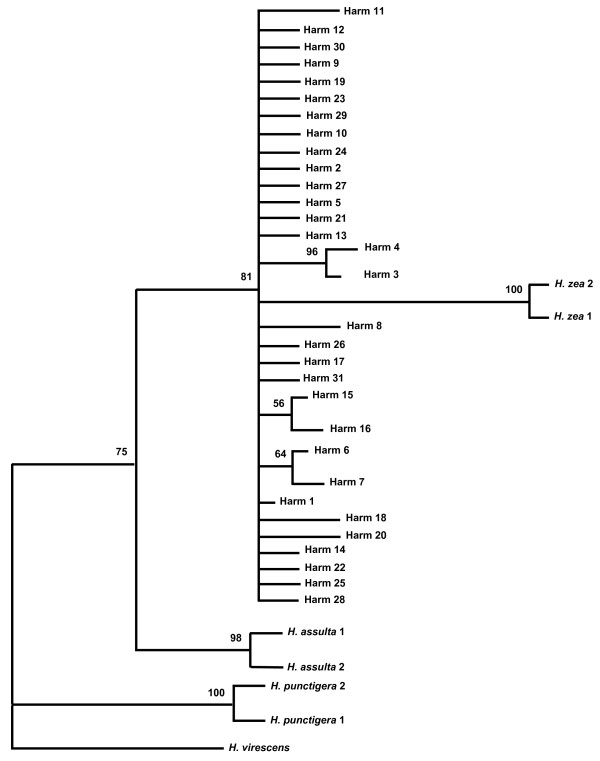
Maximum Likelihood (ML) tree of *H. armigera *(Harm-1 to Harm-31), *H. zea *(Hzea-1, Hzea-2), *H. assulta *and *H. punctigera *based on partial COI haplotypes sequences. Numbers above the nodes indicate bootstrap support. The outgroup used was *Heliothis virescens*. The inclusion of additional haplotypes Harm-32, Harm-33, and Hzea-3 to Hzea-11 did not alter the overall topology, and bootstrap values of the ML tree after 1,000 bootstrap replications remained high, with all *H. zea *haplotypes confidently clustered (bootstrap value = 96) within the *H. armigera *clade. *H. punctigera *remained basal to *H. assulta *(bootstrap value = 99), and the *H. armigera*/*H. zea *clade (bootstrap value = 78) shared a most common ancestor with *H. assulta *(bootstrap value = 97) (data not shown).

The HKY + G [34] model was selected by MODELTEST version 3.7. Model parameters estimated were based on empirical base frequencies (A = 0.3064, C = 0.1441, G = 0.1016, T = 0.4479) with no proportion of invariable sites, and a 0.0142 gamma distribution shape. The Maximum Likelihood (ML) tree from partial COI sequence was sufficiently resolved between species based on the criteria of Huelsenbeck and Hillis [35], with all nodes being supported by bootstrap values of greater than 70% (Fig. 2). The range of genetic distances of *H. armigera *within Africa (Burkina Faso and Uganda) was 0.0 – 0.02, within Australia 0.0 – 0.018, and within Asia (China, India and Pakistan) was 0 – 0.014. Genetic distances between the three continents ranged from 0.0 – 0.02. Genetic distances between all four species of the genus *Helicoverpa *and *Heliothis virescens *were also estimated (Table 5). The pairwise genetic distance between *H. armigera *and *H. zea *ranged from 0.031 to 0.047, and was intermediate between the intra- and inter-specific genetic distances in these heliothine moth species.

**Table 5:** MtDNA COI pairwise genetic distances in *Helicoverpa *and *Heliothis *species.

	*H. armigera*	*H. zea*	*H. assulta*	*H. punctigera*	*Heliothis virescens*
*H. armigera*	0–0.02				
*H. zea*	0.031–0.047	0–0.008			
*H. assulta*	0.049–0.059	0.068–0.074	0.002*		
*H. punctigera*	0.055–0.068	0.067–0.074	0.068–0.070	0.002*	
*Heliothis virescens*	0.067–0.076	0.074–0.080	0.065–0.066	0.059–0.061	**

Corrected pairwise genetic distance between *Helicoverpa *species and *Heliothis virescens *based on nucleotide substitution model (HKY+G) selected by MODELTEST 3.7. * Values based on two haplotypes from five individuals each for *H. assulta *and *H. punctigera*. ** A single individual of *Heliothis virescens *was sequenced.

## Discussion

This study presents results from the informative region of the COI partial gene in *H. armigera *and *H. zea *sampled from five different continents. Preliminary study involving a total of 73 *H. armigera *and *H. zea *found no length polymorphisms or SNPs in the tRNA-Leu gene. Furthermore, *H. assulta *and *H. punctigera *also possessed identical tRNA-Leu length as that of *H. armigera *and *H. zea*. Various studies have reported the presence of INDELs at this region from insect groups such as in the honeybees *Apis mellifera *[36] and between closely related butterfly species of *Heliconius *genus [37] and *Papilio *genus [38].

The mtDNA COI haplotype network and estimates of *F*-statistics based on the 511 bp informative region strongly suggest that the *H. armigera *mtDNA from 249 samples forms a homogeneous group of haplotypes, as expected if they consist of a single species across the portion of the geographical range sampled here. Our mtDNA COI phylogeny also revealed that all *H. armigera *haplotypes were grouped as one clade and that the long branch length of *H. zea *nested within the *H. armigera *clade (Figure. 2) possibly suggests a historical founder event from *H. armigera*. The relatively low haplotype diversity identified in *H. zea *directly reflected the limited number of specimens and populations available for this species in this study. Low genetic variation in the partial COI sequences was detected in *H. armigera*, with haplotypes Harm-1, 2, 3 and 10 making up 80 percent of genetic variation observed.

Generally, the pattern of genetic variation seen at the partial COI region of *H. armigera *is continuous and consistent between regions; that is, each haplotype, when present in two or more individuals, has a wide geographic distribution. Such a pattern is common in organisms capable of long-range movement [39]. Our mtDNA COI partial sequence data therefore supports the occurrence of long distance gene flow in this pest species, which is further supported by the low *F*_st _values and low among-group haplotype variance across all three continents. Migration in *H. armigera *may not necessarily occur only as single or multiple long-distant flights, but may also involve human-aided movements of agriculture commodities between continents. The maximum genetic distance within Asian countries (India, China and Pakistan) is 0.014 as compared to within both the African (0.020) and Australia (0.018) continents. The isozyme study of Australian *H. armigera *populations sampled across a 3,000 km study area by Daly and Gregg [12] also reported very little genetic variation (*F*_st _= 0.012). Non-significant isozyme allele frequency differences were also found between populations of *H. armigera *located on either side of the Sahara desert, thereby suggesting that geographical barriers such as the Sahara desert have not prevented long distant migration in *H. armigera *[18]. Long distant migration in *H. armigera *has been further suggested by the trapping of adult moths on Ascension Island, 2,000 km from the African coast [40] and Willis Island in the Coral Sea, 450 km off the coast of Queensland, Australia [12]. That *H. armigera *constitutes a single species across its distribution range with demonstrated long distance migration ability is further supported by the intercontinental crossing experiments of Colvin et al. [21], whereby mating between *H. armigera *from the African, Asian and Australian continents did not lead to a reduction in fecundity or offspring viability.

The mtDNA COI haplotype network (haplotype clustering patterns) and phylogenetic analysis failed to revealed specific host affiliations, both within Indian populations of *H. armigera *and across the Old World. Genetic studies of generalist phytophagous insects often reveal complexes of genetically differentiated host races or cryptic species, but the extent of genetic difference correlated with host plant association in the *H. armigera *samples available in our study is inconclusive both at the global level and more specifically within the Indian populations due to small sample sizes. Bhattacherjee [22] reported that Indian *H. armigera *showed variability in host preferences, responded differentially to parasitoid attacks [26] and when exposed to pheromone blends [25], suggesting the presence of sub-species or races. However, Jadhav et al. [41] failed to confirm the presence of sub-species in *H. armigera *based on a survey of male internal morphological characters and their association with hosts. Laboratory experiments of Kranthi et al. [29] showed that laboratory cotton and non-cotton races of *H. armigera *possessed distinct internal morphological features (numbers of cornuti on the male aedeagus). As an example of a study in which mtDNA COI sequence comparisons did reveal cryptic species, Crespi et al. [42] found that the Australian gall forming thrips represented a pair of sibling species, previously indistinguishable. Our study failed to find such differentiation based on 511 bp of the mtDNA COI gene. Currently, there is no compelling molecular evidence for the existence of *H. armigera *sub-species or races in the Old World. Further work using nuclear DNA makers or genes associated with the detoxification of secondary plant compounds may yet reveal differentiation. For example, strong genetic differentiation in maize and mugwort races of the European corn borer (*Ostrinia nubilalis*) was reported by Thomas et al. [43] based on allozyme markers, while only low genetic differentiation of races was detected by mitochondrial DNA analysis [43,44].

Phylogenetic analysis of the four important pest *Helicoverpa *species indicated that all *H. armigera *haplotypes formed a monophyletic clade which included the New World species *H. zea*. This phylogeny also revealed that *H*. *punctigera *is basal to *H. assulta*, which is in turn basal to *H. armigera *and *H. zea *(Fig. 2). The current mtDNA COI phylogeny is congruent with the phylogeny of Mitter et al. [1] as inferred from morphological characters and allozyme data, although their work did not provide a well resolved phylogenetic relationship between *H. armigera*, *H. zea *and *H. punctigera*. In this investigation the phylogenetic relationships of all four *Helicoverpa *pest species based on mtDNA COI region were sufficiently resolved and supported by bootstrap values of greater than 70%. This study demonstrated that the mtDNA COI gene is suitable for resolving *Helicoverpa *phylogeny. The inclusion of other *Helicoverpa *species and combining evidences from mtDNA COI and rapidly evolving nuclear non-coding sequences (ie, intron sequences) will be necessary for constructing a robust *Helicoverpa *phylogeny, especially that between the two closely related *H. armigera *and *H. zea *species. A monophyletic relationship between *H. armigera *and *H. zea *(with low bootstrap support values and limited sample sizes) was inferred based on the nuclear Elongation Factor 1-alpha (EF1-α) gene [45] and the Dopa Decarboxylase (DDC) gene [46], while a DDC and EF-1α multi-gene phylogeny also supported monophyly between *H. zea *and *H. armigera *with a higher bootstrap value [46].

Pairwise genetic distances between *H. armigera *and *H. zea *(0.031 – 0.047) were intermediate between that expected for intra- and interspecific comparisons, thereby indicating high levels of genetic similarity between *H. zea *and *H. armigera*. Hebert et al. [47] proposed the 3% nucleotide divergence level at the mtDNA COI gene for differentiating between different Lepidoptera species, although Whinnett et al. [48] cautioned against this and advised the inclusion of additional data types prior to drawing such conclusion. In the noctuid moth *Busseola fusca*, Sezonlin et al. [49] reported 3.1% nucleotide divergence (p distance) in mtDNA Cytochrome *b *gene between different clades of the same species. The similar genetic distance reported in this study suggests that *H. armigera *and *H. zea *are more closely related to each other than the case with the *Busseola fusca *species, possibly due to a more rapid divergence rate.

The estimates of divergence time between *H. armigera *and *H. zea*, by applying a linear rate of substitution in short term evolution [50] of 2% per million years [51], suggest that the North and South American continents *H. zea *populations were established via a founder event from *H. armigera *no more than 1.5 million years ago. This is further supported by highly similar morphology [4] and the mating compatibility between these two species [4,52,53], although the possibility of mating incompatibility via mechanical isolation (ie, locked in copula) between these two species was also reported [4]. Based on high sequence homologies in the transposable element *piggyBac*, Zimowska and Handler [54] proposed *H. zea *and *H. armigera *inter-mating as a possible explanation for the propagation of these mobile elements, while arguing for conspecific status in *H. zea *and *H. armigera *should these elements share the same genomic insertion sites in these two species. The founder event hypothesis should be further tested through a better *H. zea *sampling regime across the northern and southern American continents, as strong geographic components to intraspecific polymorphisms and inadequate sampling can lead to inaccurate phylogenies [55].

## Conclusion

This study suggests that for pest management purposes, there is currently no molecular basis for treating geographic or host specific populations of *H. armigera *differently. The current study does not suggest that *H. armigera *and *H. zea *are a single species but does show their close relationship and indicates a fairly recent (<1.5mya) divergence of *H. zea *from a parental *H. armigera *stock. We should utilise their genetic similarity as the basis of formulating strategies for concerted future research efforts into areas such as complete genome sequencing, and the mapping and identification of economically important genes (e.g., insecticide or allelochemical resistance associated genes) through crossing experiments. The scientific community is now presented with a valuable opportunity, whereby the complete genome sequencing of either one of these species will significantly advance our understanding of genome organisation of both, and thus our efforts in fighting against two of the most destructive lepidopteran pests of the Old and New Worlds.

## Methods

### Samples collection and DNA extraction

A total of 249 *H. armigera *individuals were collected from India, China, Pakistan, Burkina Faso and Uganda as larvae, adults or pupae. Larvae were collected directly from host plants or were laboratory reared first generation individuals (Table 1). Genomic DNA was also extracted from 64 *H. zea*, five *H*. *punctigera*, and five *H. assulta *adults. Phylogenetic analysis used *Heliothis virescens *as the out-group. All samples were preserved in 100% ethanol and stored at -20°C prior to DNA extraction. Genomic DNA was extracted using the DNeasy ^® ^Tissue Kit (Qiagen, cat. # 69506) or the method of Zraket et al. [56].

### Primers, PCR and sequencing

In a preliminary population survey we randomly sequenced 62 *H. armigera *(10 Australia, 10 China, 10 Pakistan, 12 Burkina Faso, 20 India), representative haplotypes of 11 *H. zea *and two of each *H. assulta *and *H. punctigera *at the mtDNA region (809 bp) that spanned the partial COI gene (552 bp) at the 3' end, the complete intergenic region between COI and COII genes (tRNA-Leu, 67 bp) and the 5' partial region of the COII gene (190 bp). Due to the general homogeneous nature of sequences at the terminal region of COI, the tRNA-Leu gene and the COII partial gene (sequence data available upon request), we restricted our population analysis to include only the informative partial COI (511 bp) region. For all remaining population samples to be analysed, a 680 bp fragment of the mtDNA COI gene was initially PCR amplified using the primers COI-F01 (5' TTATTTCACATCAGCTACTAT 3') and COI-R01 (5' CTTTATAAATGGGGTTTAAAT 3') and subsequently trimmed to the desirable 511 bp. Primers were designed based on *H. armigera *COI sequences deposited in GenBank (AY437834, AY437835, AF467260) using the primer analysis software Oligo 6.4 (Molecular Biology Insights, Ins). PCR conditions used the following profile: 94°C for 4 minutes (one cycle); one minute each of 94°C, 50°C and 72°C (35 cycles), followed by a final extension cycle of 72°C for 5 minutes. PCR amplification of individual DNA samples was carried out in a total reaction volume of 25 μL, and contained 25 ng of genomic DNA, 0.2μM each of forward and reverse primers, 0.2 mM of dNTP's, 1× PCR reaction buffer (Promega), 1.5 mM Mg^2+ ^and one unit of *Taq *DNA polymerase (Promega).

Amplicons were purified using the QIAquick^® ^PCR purification Kit (Qiagen, cat. # 8106) prior to the sequencing reaction. The DNA sequencing reaction used the ABI BigDye^® ^dideoxy chain termination sequencing system (Applied Biosystems) following the supplier's instructions, and was electrophoresed by the Australian Genome Research Facility (AGRF, Melbourne). Double stranded sequences were obtained for all samples, for accuracy. In addition, 45 individuals including representatives of each haplotype were sequenced at least twice using amplicons from separate PCR reactions to ascertain the accuracy of the single nucleotid polymorphisms (SNPs) detected. Sequences were assembled using Pregap4 and Gap4 programs within the molecular biology software STADEN package [57]. Nucleotide sequences were aligned using the sequence alignment program Clustal X [58] and were checked manually. Due to the absence of INDELs, alignment of all sequences was straight forward. Sequences that differed by one or more nucleotides were considered as different haplotypes, while sequences exhibiting identical SNPs at same nucleotide positions were considered as same haplotypes. MtDNA COI partial amino acid sequence alignment was also carried out using the sequence alignment program Clustal X.

### MtDNA haplotypes network and COI phylogenetic analysis

A mtDNA COI haplotype network for *H. armigera *was constructed manually and verified using the program TCS, version 1.13 [59]. For the maximum likelihood (ML) analysis, the computer program MODELTEST 3.7 [60] was used to determine the optimal model of nucleotide evolution. We applied ML analysis using the computer program PAUP* 4.0b 10 [61] to assess the phylogenetic relationships between the *Helicoverpa *moth species. A heuristic tree search with the 'asis' stepwise addition and TBR branch swapping algorithm was used to find the best ML tree. To assess branch support in our ML trees we used non-parametric bootstrapping with heuristic searches of 1,000 replications. We implement the criteria of Huelsenbeck and Hillis [35] to assess the confidence of tree topology, whereby nodes with confidence intervals of > 70% are considered as sufficiently resolved, while weakly resolved nodes are defined as having confidence intervals of 50% to 70%. The distance matrix option of PAUP *4.0b 10 [61] was used to calculate inter- and intra-species genetic distances as inferred from the nucleotide substitution model selected by MODELTEST 3.7.

### Statistical analysis of population structure

Genetic differentiation among all predefined *H. armigera *populations were estimated using single locus *F*-statistics of Weir and Cockerman [62] in GENEPOP v3.4 [63]. Analysis of molecular variance (AMOVA; Excoffier et al. [64]) and diversity index calculations were performed with the AREQUIN 2.000 software [65] using pairwise haplotypes distance as the distance measure. We partitioned our data for hierarchical analyses into three groups as Asia (India, Pakistan and China), Africa (Burkina Faso and Uganda) and Australia (Orbost, Dalmore and Werribee). Due to small sample sizes and non-significant *F*st values in the ten Indian populations, all ten populations were pooled in AMOVA analysis. Levels of polymorphism between countries were estimated using haplotype diversity (*h*, probability that two randomly chosen haplotypes are different in the sample) and nucleotide diversity (π) [66].

## Abbreviations

RAPD, random amplified polymorphic DNA; mtDNA, mitochondrial DNA; COI, cytochrome oxidase subunit I; COII, cytochrome oxidase subunit II; tRNA-Leu, transfer RNA-Leucin; SNPs, single nucleotide polymorphisms; ML, maximum likelihood; INDELs, insertions and/or deletions.

## Authors' contributions

DAR, BRA and KRK designed the study. KRK also provided various Indian *H. armigera *samples, DAR obtained African and Asian *H. armigera *samples and various American *H. zea *samples. Field work in Victorian was carried out by GTB. GTB carried out all the molecular genetic work and generated all sequence data. Analyses of sequence data, phylogenetic and population genetic analyses were performed by GBT and WTT. GTB and BRA worked on the mtDNA haplotype network. WTT, PB and DGH helped with interpretation of sequence data and results from population genetic analyses. GTB led the writing of this study. The original version was critically read and significant improved by WTT, DAR, DGH and PB.
